# This Plow Needs More Than One Horse to Pull It: The Need for Global Cooperation to Promote Quality

**DOI:** 10.2196/jmir.2.suppl2.e7

**Published:** 2000-09-13

**Authors:** Richard Cleland

**Affiliations:** ^1^Federal Trade CommissionUSA

## Abstract

There is global concern over the quality of health information on the Internet, especially by those who see the tremendous potential benefit of the Internet as a communications tool. The sources for lack of quality can range from the misinformed to the misguided to fraud. Global solutions will require unprecedented levels of cooperation among all the stakeholders (consumers, academia, industry, governments) as well as the best use of available technology. Quality rating systems, self-regulation and aggressive law enforcement must all play a role. One of the most formidable challenges will be how to deal with the global nature of the commerce. Who will protect consumers in foreign countries and how will they be protected? At present, the answer is not readily apparent, but complaint data collection and sharing is a critical first step.

